# How Energy Supports Our Brain to Yield Consciousness: Insights From Neuroimaging Based on the Neuroenergetics Hypothesis

**DOI:** 10.3389/fnsys.2021.648860

**Published:** 2021-07-06

**Authors:** Yali Chen, Jun Zhang

**Affiliations:** ^1^Department of Anesthesiology, Fudan University Shanghai Cancer Center, Shanghai, China; ^2^Department of Oncology, Shanghai Medical college, Fudan University, Shanghai, China

**Keywords:** (un)consciousness, cerebral metabolism, neural activity, functional connectivity, anesthesia

## Abstract

Consciousness is considered a result of specific neuronal processes and mechanisms in the brain. Various suggested neuronal mechanisms, including the information integration theory (IIT), global neuronal workspace theory (GNWS), and neuronal construction of time and space as in the context of the temporospatial theory of consciousness (TTC), have been laid forth. However, despite their focus on different neuronal mechanisms, these theories neglect the energetic-metabolic basis of the neuronal mechanisms that are supposed to yield consciousness. Based on the findings of physiology-induced (sleep), pharmacology-induced (general anesthesia), and pathology-induced [vegetative state/unresponsive wakeful syndrome (VS/UWS)] loss of consciousness in both human subjects and animals, we, in this study, suggest that the energetic-metabolic processes focusing on ATP, glucose, and γ-aminobutyrate/glutamate are indispensable for functional connectivity (FC) of normal brain networks that renders consciousness possible. Therefore, we describe the energetic-metabolic predispositions of consciousness (EPC) that complement the current theories focused on the neural correlates of consciousness (NCC).

## Introduction

Our brain is energy hungry and consumes about 20–25% of the energy of the whole body despite representing only 2% of the body mass. Most of this energy, around 80–90%, is spent for its spontaneous activity, as measured in the resting state, that is, without any specific task demands. However, there is only an incremental increase, up to 10% in energy, for specific tasks (Shulman et al., 2014). The exact relevance of such a high energetic-metabolic consumption by the brain, as well as its relevance to behavior, remains unclear. Few studies on sleep (Rempe and Wisor, 2014), anesthesia (Laaksonen et al., 2018), and vegetative state/unresponsive wakefulness state (VS/UWS) (Garcia-Panach et al., 2011) have shown decreased glucose metabolism in various brain regions and/or networks. These findings indicate that an unconscious state has a distinct energetic signature from an awake state. Therefore, neuroenergetics may play a crucial role in consciousness (Shulman et al., 2009).

Large-scale study of consciousness is becoming a promising framework to understand how consciousness arises from structural and functional interactions between the neural assemblies. An unconscious state show that the functional connectivity (FC) decreases between and within different brain networks (Hudetz, 2012; Demertzi et al., 2014; Altmann et al., 2016), such as the default-mode network (DMN), attentional network, salience network, central-executive network, and sensory networks, during sleep, anesthesia, or VS/UWS. Several recent studies demonstrate that the decreased FC (Stender et al., 2014, 2015, 2016) or glutamate turnover (Sibson et al., 1998; Patel et al., 2017) goes along with the decreased glucose metabolism, which provides the best predictor of levels of consciousness. This suggests that cerebral metabolism as a function of network activity is associated with consciousness.

**Figure 1:**
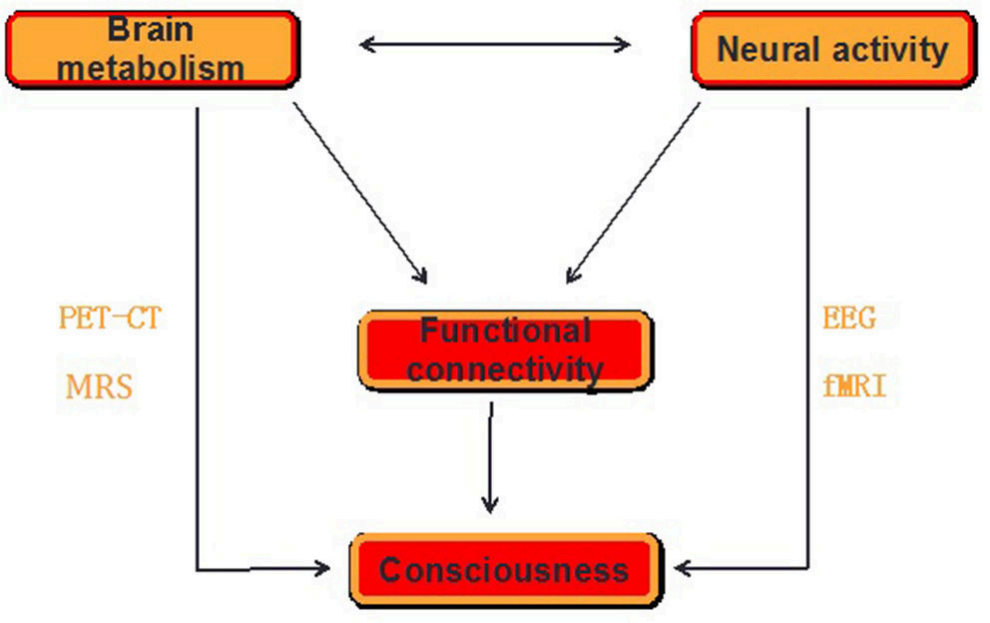
The schematics for the proposed mechanism of consciousness. Consciousness requires a form of neural activity that is metabolically expensive while the loss of consciousness is related to an uncoupling between the neural activity and brain energy, which can be measured by functional neuroimaging [PET-CT and functional MRI (fMRI)] and neurophysiological [electroencephalogram (EEG)] and neurochemical [magnetic resonance spectrum (MRS)] techniques in the form of functional connectivity (FC) within and between the brain regions. The pattern of FC is believed to be related to the level of consciousness.

Although the neural correlates of consciousness (NCC) remain unknown, several neuroscientific theories of consciousness, including the information integration theory (IIT) (Tononi et al., 2016), global neuronal workspace theory (GNWS) (Dehaene and Changeux, 2011), and temporospatial theory of consciousness (TTC) (Northoff and Huang, 2017), have been proposed. Nevertheless, neither of these theories consider the energetic basis of consciousness. Thus, we are confronted with a gap between neuroenergetics and neural mechanisms in our current account of consciousness. To close this gap, the specific aims of this paper include the following: (1) a review of how the basic metabolic-energetic processes of the brain are related to FC as it features neural activity during consciousness; (2) demonstrating how an unconscious state leads to changes in both energy metabolism and FC; and (3) discussing how neuroenergetic mechanisms potentially involve the current three theories of consciousness. In this study, we conclude that the suggested neuroenergetic hypothesis complements the current neuroscientific theories of consciousness (Figure 1), and therefore possibly providing novel insights informing future consciousness research.

## Brain Metabolism, Neural Activity, and Consciousness

Cerebral metabolism is the sum of chemical processes, which provides the energy needed to carry out various brain functions. As the brain energy consumption is linearly related to the number of neurons at work, evolutionary strategies in brain size reveal that metabolism exists as a relevant player in information processing (Fonseca-Azevedo and Herculano-Houzel, 2012). The disproportionally high-energy demands of the brain strongly suggest that its extremely high metabolic rate is related to complex neural processing.

### Energetic Metabolism in Molecular and Cellular Levels

**Figure 2:**
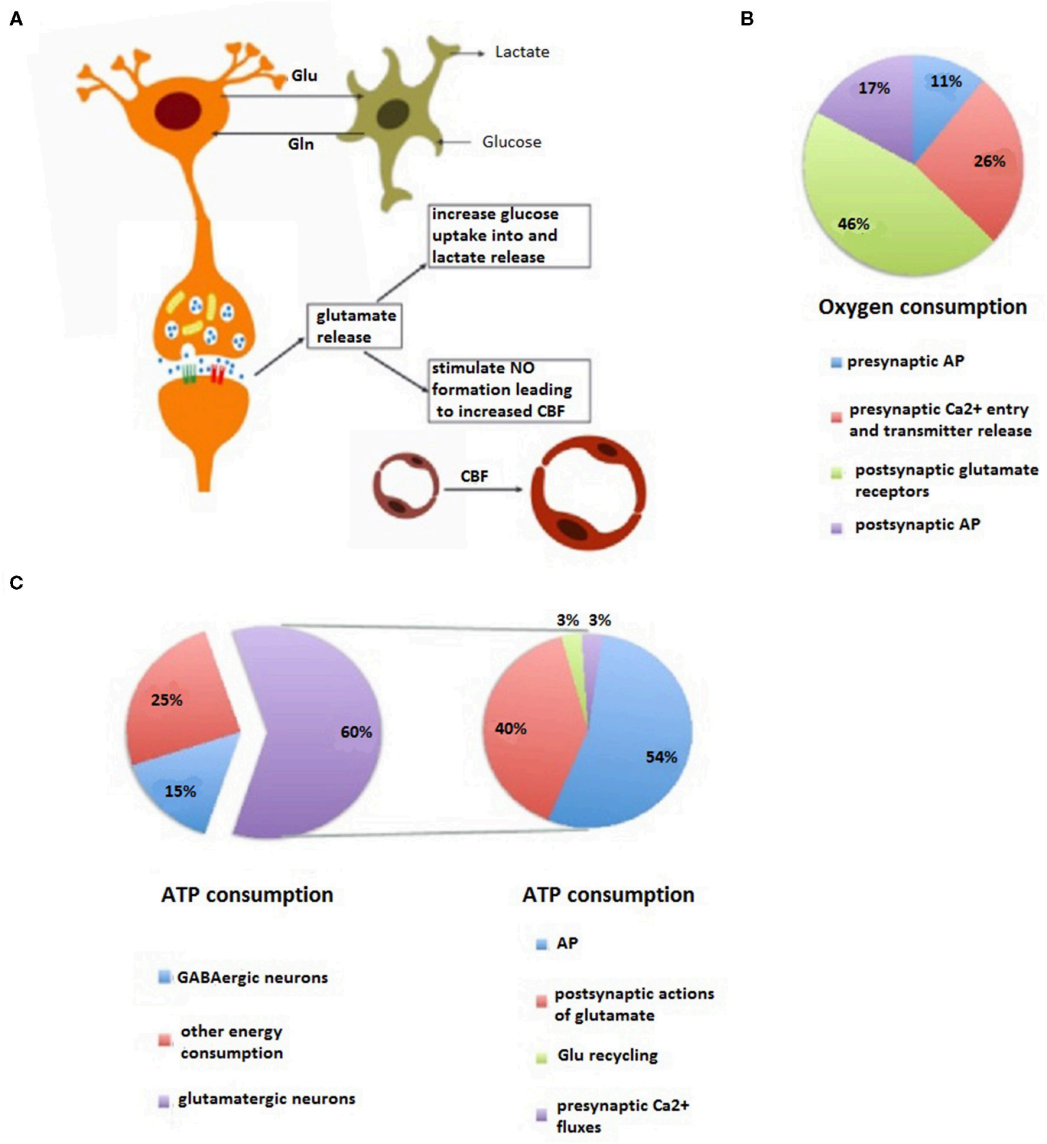
Energetic and oxygen costs and coupling of neural processing. **(A)** Neurovascular and neurometabolic coupling. Physiological changes in the neural activity of the brain are invariably accompanied by changes in local blood flow and glucose utilization. During the modality-specific activation of a brain region, glutamate release from active synapses induces the increase of glucose use and lactate production of perivascular astrocytes and an increase in cerebral blood flow (CBF) through receptor-mediated nitric oxide (NO) formation. These activity-dependent processes help neurons to meet their energy needs. **(B)** Pharmacological analysis reveals an energy budget in which 11% of O_2_ use is on presynaptic action potentials (APs), 17% is on presynaptic Ca^2+^ entry and transmitter release, 46% is on postsynaptic glutamate receptors, and 26% is on postsynaptic APs, is approximately accordance with theoretical brain energy budgets. **(C)** Most of neurons and synapses (90%) in the cerebral cortex are either glutamatergic or gamma-aminobutyric acid-ergic (GABAergic) while 75% of the total cortical energy consumption is coupled to glutamate (60%) and GABA neurotransmitter cycling (left). Specially, the energy consumption used for glutamatergic neurotransmission (right) includes various neuronal components underlying the neural activity: presynaptic AP, entry of presynaptic Ca^2+^ ions, neurotransmitter recycling (release, uptake, conversion, and storage in vesicles), and postsynaptic AP.

Metabolically, the brain is highly active and consumes glucose as it is an almost exclusive energy source. This metabolic pattern relies on a constant supply of substrates *via* cerebral blood flow (CBF) to maintain a stable energy flow, which helps the brain to meet its metabolic demands imposed by neuronal activation. This can, for instance, be measured by functional neuroimaging on a macroscopic scale (Shulman et al., 2004) or by fluorescence microscopy on a microscopic scale (Loaiza et al., 2003). High-speed and high-resolution optical imaging systems have enabled the decoding of how neuroenergetics support information processing through one neural circuitry (mesoscopic scale) related to consciousness. The neuroenergetic oscillation is neural activity-dependent, laying the foundation of functional neuroimaging (Buxton, 2021). Numerous studies provide evidence of neurovascular and neurometabolic coupling, as reflected by a linear linkage between neural activity and metabolic fluxes for adequate energy supply (Figure 2). Two models have been adopted to interpret a neurometabolic coupling (Moreno et al., 2013): the metabolic feedback model, in which energy metabolites such as adenosine and lactate in the brain couple CBF to energy consumption or neuronal signaling; and the neurotransmitter-mediated feedforward model, in which metabolites released from neuronal cells as a consequence of the glutamatergic or gamma-aminobutyric acid-ergic (GABAergic) neurotransmission contribute to a neurovascular coupling mechanism. To meet local energy demands in the synapse, metabolic gene expression (Bas-Orth et al., 2017), ATP production pathways (Ashrafi and Ryan, 2017), and metabolic compartment forms localized to synapses (Jang et al., 2016) are shaped to adapt neuronal energy levels upon synaptic activation. These mechanisms ensure adequate spatial and temporal energy flow to operate normal synaptic transmission when neurons undergo rapid and repetitive changes in firing rate (Petit and Magistretti, 2016).

Most intracellular ATP (70–80%) consumed by neurons is used to establish electrochemical gradients and actively maintain resting-membrane potentials critical for neuronal excitability. These costs are minimized by myelination but increased as a function of axonal length and diameter, with longer-distance connections being metabolically more expensive to maintain (Mukhtarov et al., 2011). The mechanisms through which neuroenergetics is integrated with neural activity and brain function have been reviewed in detail (Lujan et al., 2016). The metabolic pathway for task-demanding ATP production is highly controversial. A recent study shows that metabolically demanding tasks, such as vesicle recovery after depletion, are fueled by oxidative phosphorylation rather than glycolysis, as shown in a previous study (Sobieski et al., 2017). On the other hand, astrocytes also play an important role in binding energetic metabolism with neural transmission. Astrocytes shuttle glucose- and glycogen-derived lactate to neurons, providing energy to the brain and enabling neuronal activity and behavioral responses; the abnormal glutamate metabolism in astrocytes can directly contribute to neuronal hyperexcitability and neural network dysfunction. In turn, astrocytes are under neuronal control through specific neurotransmitter receptors, thereby regulating the states of “rest” and “activation” metabolically *via* the astrocyte–neuron interaction (Barros et al., 2018).

### Energetic Metabolism and Synaptic Transmission

Glucose also provides substantial intermediates for the *de novo* synthesis of major neurotransmitters through the glutamate/GABA-glutamine cycle. Linkage of fluxes between glutamine and glutamate/GABA with mitochondrial enzymes and oxidative phosphorylation has been used to explain neuron–astrocyte metabolic interactions involving these neurotransmitters (Patel et al., 2014). As a result, a large fraction of glucose metabolism is directly coupled to synaptic activities (Figure 2). As more than 90% of cortical neurons and synapses are either glutamatergic or GABAergic, correspondingly, 75% of the energy utilization in the whole cerebral cortex is coupled to the cycling of glutamate and GABA (Hyder et al., 2006; Rothman et al., 2011). It is known that both excitatory pyramidal neurons and inhibitory GABAergic interneurons, and their synaptic interactions, are pivotal elements for information processing as evidenced by excitatory-inhibitory components mediating functional MRI (fMRI) blood oxygen level-dependent (BOLD) responses (Kapogiannis et al., 2013) and regulating thalamocortical FC in human and rodent brains (Just and Sonnay, 2017). Presynaptic terminal ATP depletion is known to impair synaptic transmission by decreasing the number of functional synaptic vesicle release sites and slowing the refill of the vesicle pool (Du et al., 2008; Rajendran et al., 2016; Lucas et al., 2018). Furthermore, deficits in energy supply possibly lead to an abnormal excitation-inhibition balance (EIB) in neural networks and thus leading to information processing dysfunction.

Since the glutamatergic neurons have a greater neurotransmitter cycling flux than the GABAergic neurons (~4:1), cortical glucose oxidation mostly reflects the energetic cost of glutamatergic transmission (Nugent et al., 2015). Shulman et al. found a linear relationship, using a ^13^C magnetic resonance spectrum (MRS) technique, between the neural activity (reflected by glutamatergic flux) and metabolic activity (reflected by the rate of glucose oxidation) in both a non-activated status (Shulman et al., 2014) and an intensive activated status (Patel et al., 2004). Recent studies also revealed neuronal firing rates coupled with glucose consumption at different levels of consciousness in rats (Du et al., 2009) and a linear linkage between neural activity and glucose metabolism in the human cortex (Hyder et al., 2013).

### Neuroenergetics and FC

As the energetic demands related to synaptic function reflect the neural activity, PET and fMRI scans detect the signals that reflect neural activity-dependent energy consumption rather than directly measuring synaptic activity. Interestingly, a recent study has revealed that the resting-state functional networks have distinct energetic signatures, consistent with their functional specialization (Shokri-Kojori et al., 2019); for example, the frontoparietal cortex is metabolically costly, primarily coupled to its higher-order cognitive ability. When its metabolism is reduced slightly, consciousness could be lost. This lays forth the questions leading to further study on consciousness from the perspective of neuroenergetics and its transformations in relation to information processing (Strelnikov, 2014). A rapid progress in understanding neuroenergetics in the last decade has prompted us to search for the common underlying principles. Several investigators have attempted to uncover the mechanisms underlying information processing in view of brain energetic states; for example, gamma oscillations critical for a higher function of the brain positively correlate with cortical glucose metabolism (Nishida et al., 2008), and are abolished by an impaired mitochondrial function (Kann et al., 2011). Although the mechanism underlying energetics homeostasis dysregulation leading to the disruption in connectivity remains unknown, several studies have suggested that voxels with a greater degree of connectivity have greater energy demands (Thompson et al., 2016; Tomasi et al., 2017), suggesting that the energy demand scales have several direct functional connections.

The information processing requires a precise spatial and temporal coordination of neural activities (Liu et al., 2015). This coordination relies on not only anatomical but also FC among brain networks (Liu and Duyn, 2013). The different FC networks play key roles in enabling a coordinated synchronous activity concerned with the normal brain functions whereas a widespread loss of FC is thought to be associated with unconsciousness. Local neural activity is closely coupled to neural action potential (AP)/local field potential (LFP) power and FC from BOLD fMRI amplitude, which can be measured and clustered into networks. In contrast, fluctuations in the fMRI BOLD signal are closely linked to the changes in connectivity states (Maandag et al., 2007). Therefore, once neuroenergetic demands decrease, neuronal firing rates tend to slow frequencies and result in shorter-distance cortical connectivity. As the highest synchronized fluctuations in cerebral metabolic rate of glucose (CMRglc) are basically identified as homologous cortical regions (Sanabria-Diaz et al., 2013), it is assumed that the brain regions, which are significantly correlated CMRglc, are functionally associated. Therefore, when the changes in energy metabolism occur in a single brain region, other brain regions with which these are linked will also be affected correspondingly. FC maps from the cerebral metabolic rate of oxygen (CMRO_2_) signal based on fMRI are similar to those from BOLD (Rodgers et al., 2016). A simultaneous PET/fMRI study in healthy subjects reveals that the resting-state FC is determined by local glucose metabolism or local activity within and across DMN (Passow et al., 2015). Meanwhile, the pattern of glucose metabolism is closely associated with the spatial pattern of increased FC (Riedl et al., 2014).

Being an identical source, neuroenergetics may also govern the network topology of all synaptic connections. The core of those networks, such as the DMN, tends to have high levels of CMRglc (Bennett et al., 2015), displaying a significantly higher level of aerobic glycolysis (Riedl et al., 2016). As a result, a significantly heterogeneous pattern of correlations with PET is found across both functional networks and anatomical regions with the strongest correlation for DMN (Vaishnavi et al., 2010). The brain networks generally have self-organizing small-world properties of high clustering and high global efficiency, indicating several highly connected hubs (“rich club”). The pattern of “rich clubs” seems to support both segregated and integrated information processing and enables faster, more direct, and less noisy information transfer (Aiello et al., 2015). As high energetic costs associated with neural structures favor energy-efficient coding and wiring schemes (Sporns and Kotter, 2004), this results in a high vulnerability to metabolic stress. Thus, we might expect the most metabolically expensive nodes and edges to be particularly vulnerable to disrupted connectivity. Small-worldness is also found to exist in the domain of metabolic networks (Hu et al., 2015). Metabolism is hierarchically ordered, extending across both spatial and temporal domains, and modular, related to interactions between anatomical and functional modules at different levels of complexity (Bullmore and Sporns, 2012). The modular nature of metabolism may be explained by the fact that such interactions subside progressively as distances grow. Therefore, the more frequently FC patterns inherit anatomical connectivity, fewer small-world properties are seen under anesthesia (San Martin et al., 2014), sleep (Barttfeld et al., 2015), or conscious disorders (Lv et al., 2015). As “rich clubs” commonly identified in the regions belong to the DMN and attentional network (Chennu et al., 2014), these findings highlight the vulnerability of these higher metabolically dependent “rich clubs” to pharmacological and pathological changes in energy resources. We assume that once the “rich club” organization is destroyed, the energy threshold associated with state transitions increases significantly since the economic mode of energetic metabolism supporting small-worldness would not be maintained anymore. These changes in topological and energy efficacy may play an important role in consciousness.

In sum, the neural activity and glutamatergic neurotransmission are highly energy demanding and coupled to glucose oxidation. The metabolic state in the brain, specifically in “hubs,” would profoundly affect network excitability and connections. It is expected that a higher energetic level drives more structured and organized networks with a longer-distance FC. Nevertheless, hypermetabolism in focal brain regions/networks is not necessarily associated with an elevated FC and a higher conscious level. For example, although a coupling between the energy consumption and neural activity within a gray matter is reported in epileptic status; however, this metabolic synchronization is spatially heterogeneous throughout the brain, in the regions showing hypermetabolism and an increased FC, generation and propagation of epileptiform activity usually occur; in contrast, in the regions partly located in the areas corresponding to the DMN, showing hypometabolism and an decreased functional activity, information communication is interrupted (Wang et al., 2020), suggesting that widespread, asymmetric, and often severe interictal metabolic alterations play a critical role in epileptic loss of consciousness. In other words, a neurometabolic decoupling in neuroglial populations might play a role in a higher coupling, which may be the modulating result linked to the strong hyperexcitability generated by epileptiform activity. In a nutshell, neuroenergetics is essential to maintaining brain network homeostasis and, consequently, consciousness.

## Brain Metabolism and Loss of Consciousness

As general anesthesia typically induces a 55% reduction in the whole-brain glucose metabolism in humans (Laaksonen et al., 2018), non-rapid eye movement (NREM) sleep a 23% reduction (Guye et al., 2010), and coma a 60% reduction (Buchsbaum et al., 1989), Shulman proposes that the global energy reduction is responsible for the loss of consciousness (Shulman et al., 2009), and that the measurement of global CMRglc could be used as an approach to discriminate a conscious state from an unconscious state. Furthermore, this study revealed that anincrease in levels of consciousness is likely to be associated with a stepwise doubling of CMRglc (Hyder et al., 2013), suggesting the transition between the conscious states, and showing that metabolic pattern transitions matter. As the increments in cerebral metabolic requirements to sustain consciousness are surprisingly small (Stender et al., 2016), small decrements in the cerebral metabolic supply may be sufficient to alter conscious state of an individual. Therefore, the interventions targeting neuroenergetics may change the conscious state. In this study, we provide an overview of the relationships between the energetic metabolism, neural activity, and FC during sleep, anesthesia, and VS/UWS status.

### Sleep

Sleep is a nearly ubiquitous phenomenon across a broad range of mammalian species. Unlike those seen during wakefulness and rapid eye movement (REM) sleep, neural activities during NREM sleep generally decrease (Levy et al., 1987), and cortical neurons continuously and synchronously cycle between the synchronized firing periods (UP states) and silent periods (DOWN states), corresponding to electroencephalogram (EEG) slow-wave activity (SWA). In contrast, the EEG signal during REM sleep is similar to that during a waking state as it is characterized by oscillations at low amplitude and high frequencies.

#### Brain Metabolism and Sleep

Non-rapid eye movement is characterized by a decrease in cerebral metabolism and an increase in slow waves. A previous animal study suggested that energy participates in slow-wave sleep (SWS), per changes in the extracellular concentrations of glucose in the cerebral cortex during a sleep/wake cycle (Dash et al., 2013). Altered cerebral metabolism, including the major metabolic pathways during the transition from wakefulness to sleep, has been reviewed elsewhere (Aalling et al., 2018). Briefly, the difference in neuronal firing pattern during NREM sleep or REM sleep and wakefulness is positively correlated to its metabolic rate. Given the association between sleep/wake cycles and profound shifts in cerebral metabolism, PET studies clearly show that CMRglc is reduced globally by 12–44% and CMRO_2_ by 25–40% in NREM compared to wakefulness (Madsen et al., 1991). In a regional level, SWA during NREM promotes a decline in glycolysis in the cerebral cortex (Wisor et al., 2013). From a cellular level, neuronal silence leads to a decline in the number of APs and demands for ATP-dependent membrane repolarization (Attwell and Laughlin, 2001). However, this relationship does not apply to REM, an energy state in the cerebral cortex that is being active as in a waking state. Furthermore, Katayose et al. pointed statistically greater energy expenditure in REM relative to NREM stage 2 (N2) or stage 3 (N3) sleep (Katayose et al., 2009), in accordance with the previously reported significant differences in metabolic rate between REM and N2 (REM > N2), and between N2 and N3 (N2 > N3) (Fontvieille et al., 1994).

#### FC During Sleep

Growing evidence has suggested that conscious levels during sleep can be reflected in FC patterns, which allows us to classify sleep stages with an FC analysis. Sleep has been repeatedly shown to influence the magnitude of synchronization between the brain regions and disrupt the spatial organization of functional networks, but largely preserve the connectivity within sensory cortices.

Recent EEG-fMRI studies, during NREM sleep, found that the FC in frontoparietal and thalamocortical networks decreased while they remained preserved or increased in unimodal sensory cortices (Braun et al., 1997; Larson-Prior et al., 2011) similar to the findings of PET studies (Kajimura et al., 1999; Nofzinger et al., 2002). Dynamic FC also consistently differentiates EEG-directed wakefulness and NREM stages (Samann et al., 2011). Further, fMRI resting-state connectivity analyses have suggested that the DMN may play a key role in maintaining conscious awareness, as the sleep-induced alteration of consciousness levels is reflected in the correlation between the DMN components (Zhou et al., 2019). Meanwhile, the functional integrity of the DMN may distinguish deep sleep characterized by a functional uncoupling of the DMN from REM sleep characterized by a recoupling of the DMN (Horovitz et al., 2009). With deepening sleep, changes in connectivity involving the DMN subsystems, such as the posterior cingulate cortex (PCC)/retrosplenial cortex (RspC), parahippocampal gyrus, and medial prefrontal cortex (mPFC), can define the different stages of sleep (Chow et al., 2013). Another study found that significantly increased connectivity of the middle and inferior occipital gyri, including calcarine gyrus, lingual gyrus, and cuneus, to brain regions were observed in NREM sleep as compared to wakefulness, alongside decreased connectivity between the calcarine gyrus with lingual gyrus and cuneus (Altmann et al., 2016). These results indicate that the failure of input information to propagate to frontoparietal networks triggers the loss of conscious perception during sleep.

### Anesthesia-Induced Loss of Consciousness

Anesthesia is not the same as sleep although general anesthetics may act on neural circuits regulating wake/sleep cycles. There is a large difference in the neurovascular coupling and brain-wide circuit function between an animal under anesthesia and those in the awaken state. General anesthetics may induce reversible unconsciousness by inhibiting the glutamatergic or enhancing the GABAergic transmission. It is thought that general anesthetics with distinct pharmacological properties affect both spontaneous and evoked neuronal fire rates in one way or another, pointing to a consciousness-related effect distinct from a drug-related effect. *In vitro* studies found that volatile anesthetics and propofol selectively inhibited the basal and evoked release of glutamate from cortical nerve terminals, alongside enhanced or unaffected GABA release (Westphalen and Hemmings, 2006), suggesting that the disruption of EIB inhibits the information transfer between brain regions/networks. Therefore, one may postulate that anesthetics interfere with brain energy homeostasis, including alterations in spatiotemporal firing patterns, a reduction in the neural metabolic activity below the threshold required to support neural signal transmission across the whole brain, and an interruption in neural computing sufficient to induce the loss of consciousness.

#### Effects on Cerebral Metabolism

**Figure 3:**
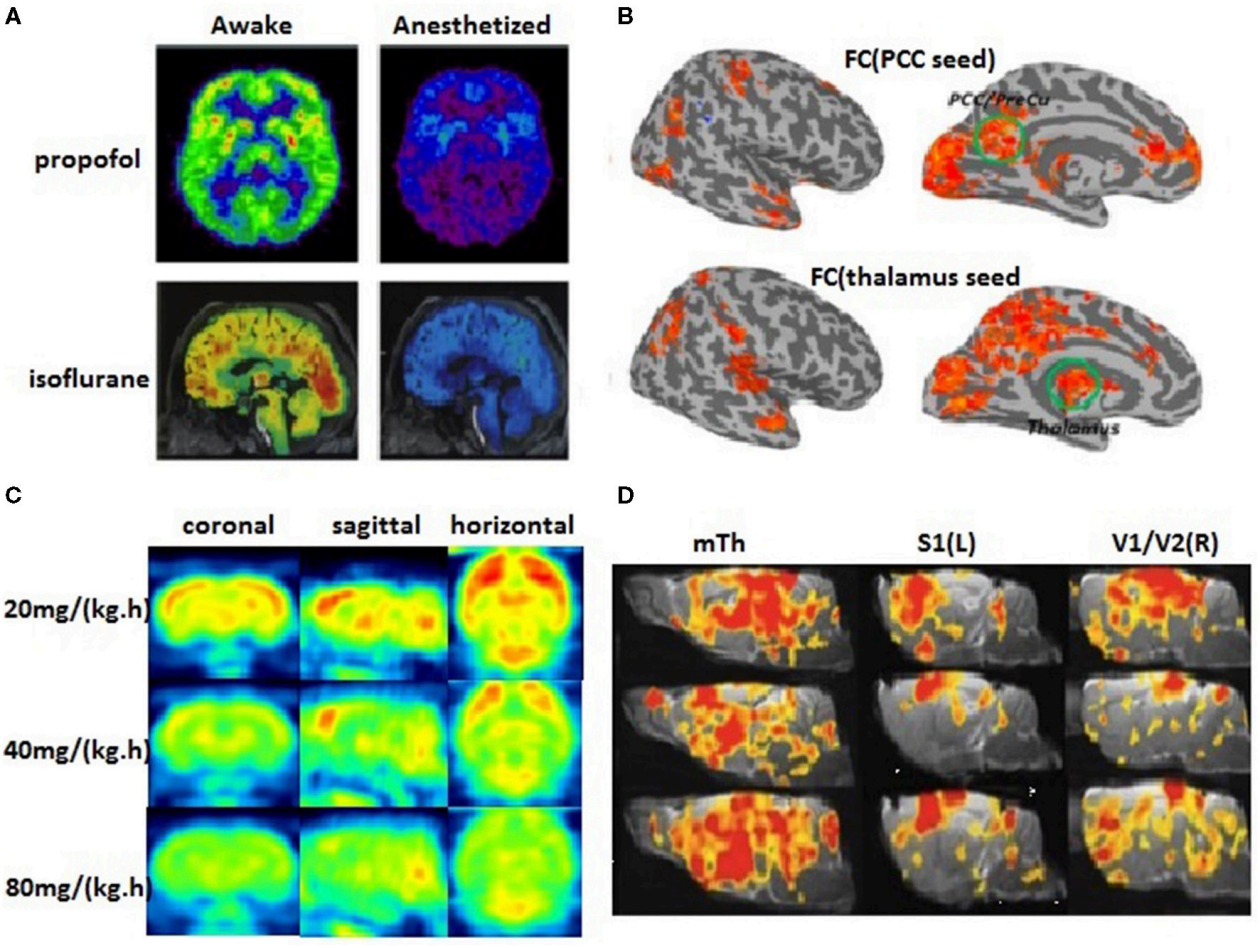
The cerebral metabolic rate of glucose (CMRglc) and FC. **(A)** The PET scans are compared between awakefulness and uncosciousness induced by general anesthetics (Nielsen et al., 2013). Red areas demonstrate the most significant findings and blue/purple the least, yet significant findings. **(B)** Group comparison of the FC with two seed regions in the posterior cingulate cortex (PCC) and the thalamus showed widespread reduced corticocortical and thalamocortical connectivity in the anesthetized state (Huang et al., 2014). **(C)** The metabolic rate of glucose in rat brains decreases correspondingly with the gradually deepened level of propofol anesthesia after intravenous infusion of 20, 40, and 80 mg/kg/h propofol (our unpublished data). **(D)** Dose-dependent change of brain FC in rat brains (Liu et al., 2013).

It is generally accepted that general anesthetics significantly depress synaptic field oscillations and firing rates by reducing cerebral metabolism. The decline in CMRglc has been observed in all major brain regions, especially in the thalamus and the midbrain reticular formation during sevoflurane, propofol, dexmedetomidine, or xenon anesthesia (Rex et al., 2006; Schlunzen et al., 2010, 2012; Akeju et al., 2014). Propofol produces a greater effect on forebrain structures than on hindbrain structures while the single largest regional change is found in the cingulate cortex (Dam et al., 1990). Isoflurane anesthesia results in CMRglc being spread out more uniformly across the brain compared with the awake state (Alf et al., 2014) (Figure 3). Interestingly, an fMRI analysis in rats revealed a decrease in cortical connectivity before subcortical connectivity (Liu and Duyn, 2013). This spatial or temporal heterogeneity in metabolic changes caused by anesthetics implies disruptions in FC and a higher-order cortical information integration.

Anesthesia-induced reduction in cerebral metabolism may be related to mitochondrial dysfunction. Several experimental studies have shown that volatile anesthetics and intravenous anesthetic propofol depolarized neural mitochondria and inhibited the ATP synthesis in both rat and human nerve terminals by directly inhibiting respiratory chain complex I activity (Bains et al., 2006, 2009), which sufficiently causes ATPase reversal. The greater inhibition of mitochondrial-dependent ATP synthesis by increasing the depth of anesthesia and a stronger depression in the neural activity reflected by the EEG power reveal a tight neurometabolic correlation between the oxidative ATP production rate and the brain activity level. Therefore, it is not surprising that those non-anesthetic agents or hypoglycemia, leading to decreased intracellular ATP levels, can enhance anesthetic effects (Wu et al., 2002; Wang et al., 2015). Furthermore, mitochondrial defects increase the sensitivity to volatile anesthetics from nematodes to humans carrying complex I mutations by inhibiting energy-dependent excitatory neurotransmission, including vesicle endocytosis and exocytosis (Zimin et al., 2016; Olufs et al., 2018). As neuroglia units control the energy supply of brain, astrocytic metabolism impairment is also likely to disrupt the energy resources and thus synaptic transmission (Sonnay et al., 2017). A recent study has found that an astrocyte-specific *Ndufs4* knockout mouse was impaired in its emergence from general anesthesia (Ramadasan-Nair et al., 2019). As a result, transgenic mice lacking a subunit of mitochondrial complex I *Ndufs4* are extremely hypersensitive to volatile anesthetics and propofol (Quintana et al., 2012). Interestingly, the loss of the subunit of mitochondrial complex I in a subset of glutamatergic neurons promotes isoflurane hypersensitivity while its loss in GABAergic or cholinergic neurons does not (Zimin et al., 2016).

Furthermore, knockdown of the mitochondrial complex I in regional brain areas (central and dorsal medial thalami and parietal association cortex) also increases the sensitivity to volatile anesthetics, suggesting that neuroenergetic capacities in the thalamocortical circuit are likely to be an important determinant for the maintenance of consciousness. Intracerebral microdialysis also showed that sevoflurane-induced mitochondrial dysfunction was experienced at the very beginning of the administration, marked by an increase in cerebral lactate and lactate/pyruvate ratio (Ramadasan-Nair et al., 2017). In contrast, another intravenous anesthetic, ketamine, is associated with an increase in the utilization of cerebral glucose. Evidence suggests that the effect of *Ndufs4* knockout may be indirect, affecting an energetic state necessary for ketamine-induced sedation (Nielsen et al., 2013). Ketamine has also shown altered activity of the mitochondrial respiratory chains in several rat brain regions, including the prefrontal cortex, striatum, and hippocampus (Carspecken et al., 2018). Further analyses showed that ketamine dose-dependently inhibited respiratory chain complex I activity, which reduced oxidative phosphorylation efficiency and ATP synthesis during unconscious periods (de Oliveira et al., 2011). Ketamine also controls the glutamate release by compromising the activity of nicotinamide adenine dinucleotide phosphate (NADPH) oxidase, one of the respiratory complexes (Sorce et al., 2010). These findings support the fact that consciousness is susceptible to altered neuroenergetic homeostasis.

General anesthesia also suppresses neuronal glucose uptake whereas sensory stimulation, a method to increase information input, leads to a sharp increase in neuronal glucose uptake (Rajendran et al., 2016). Exocytosis inhibition by isoflurane in glutamatergic nerve terminals was greater than the inhibition in GABAergic nerve terminals, consistent with the selective inhibition of excitatory synaptic transmission during anesthesia (Baumgart et al., 2015). Such a disruption of EIB might mediate reduced interactions between neurons and the observed network-selective effects during anesthesia.

#### Anesthetics-Induced Disruption in Functional and Metabolic Connectivity

Unlike in an awake condition, general anesthetics decrease the amplitude and increase the lag in the BOLD neural signals (Gao et al., 2017). By comparing the neural activity between an awake state and general anesthesia-induced loss of consciousness, the NCC can be investigated by functional neuroimaging techniques. For instance, sevoflurane significantly altered resting-state (Qiu et al., 2008a) and task-induced (Qiu et al., 2008b) region-specific CBF-BOLD coupling even at a low concentration (0.25 minimum alveolar concentration). In anesthetic-induced sedation states, the strength of the FC in key nodes of the frontoparietal network and midline DMN, as well as in thalamocortical networks, show a linear correlation with a lower conscious level. While the propofol-induced loss of consciousness greatly reduces cortico-cortical connectivity, especially in the frontoparietal network and midline DMN (Boveroux et al., 2010), accompanied by decreases in CMRglc of about 50–60% in different areas of the cortex, this suggests a causal relationship between reduced metabolic activity and altered FC. Being consistent with the results in mitochondrial mutants, impeding mitochondrial function can impact the resting-state FC (Sanganahalli et al., 2013).

Recently, metabolic connectivity (MC, coherence between glucose metabolism in different brain regions) is introduced based on the concept that most of the total energy required for neuronal communication is consumed at the target neurons. A previous study has shown that MC strength linearly increased with CMRglc, implying that the MC measurement is strongly associated with the energy demands of the brain. Several studies have investigated the effects of anesthetics on MC. Meanwhile, White and Alkire showed that CMRglc in the human thalamus region was significantly suppressed under isoflurane anesthesia (White and Alkire, 2003). In a recent study (Chen et al., 2019) using the resting-state fluorodeoxyglucose- (FDG-) PET data from the rat brain, we found a certain degree of heterogeneous propofol-induced glucose consumption reduction throughout the brain, accompanied by the changes in a spatial distribution pattern of brain metabolism, that is, from a hierarchical to a more uniform metabolic pattern across the whole brain. Moreover, a graph-theoretic analysis showed a breakdown of the metabolic network characterized by a decrease in MC and network global/local efficiency during propofol-induced deep anesthesia in comparison to a sedation state. Another PET study also suggested that MC impairment primarily involved thalamocortical and corticocortical projections in an unconscious state (Vespa et al., 2005). Using 2-deoxyglucose imaging data in mice, Dawson et al. found that an acute ketamine treatment increased metabolic activity in the prefrontal cortex but decreased metabolic activity in the dorsal reticular thalamic nucleus; this is associated with abnormal FC between the PFC and multiple thalamic nuclei (Dawson et al., 2013). These results suggest that local metabolic activity is closely linked to brain FC, suggesting that neurometabolic inhibition by general anesthetics is directly related to aberrant network connectivity and thus resulting in loss of consciousness.

### Disorders of Consciousness (Minimal Conscious State and VS/UWS)

Coma is commonly seen in patients with a traumatic brain injury (TBI). Experimental and clinical studies suggest that metabolic perturbations could occur immediately after TBI, including in the form of a deficiency in substrate supply, mitochondrial dysfunction, and an increase in metabolic demand. This metabolic perturbation may be detected by using PET and MRS techniques in patients with TBI or microdialysis in subarachnoid hemorrhage individuals. Notably, the PET data showed that CMRglc in VS/UWS, a severe type of disorders of consciousness (DOC), was reduced to 40–50% (Laureys et al., 2004). In patients with chronic severe TBI, those with a higher glucose uptake are associated with a higher level of wakefulness and better neurological outcomes (Yamaki et al., 2018). Furthermore, energy consumption levels in VS/UWS extract no more than 34% of the normal average; in contrast, the minimal conscious state (MCS), a less severe type of DOC extracts close to 50% of the normal average (Garcia-Panach et al., 2011). These findings suggest that DOCs can be considered as pathological neuroenergetic disorders.

#### Metabolic Signatures of FC in DOC

A significant link between the severity of DOC and global hypometabolism unrelated to cerebral ischemia has been described in an increasing number of studies (Hattori et al., 2003). This hypometabolism in brain structures is mostly related to anatomic and functional disconnections: direct focal lesions, white matter damage due to diffuse axonal injury, or remote disconnection/deafferentation (Bruno et al., 2012). As a result, the degree of altered connectivity may be related to the severity of impaired consciousness. Furthermore, the changes in glucose metabolism in various cortical structures are shown to be linked to the corresponding neurological dysfunction, including memory, cognition, and consciousness; the most severe condition (VS/UWS) is characterized by the most severe form of hypometabolism (Garcia-Panach et al., 2011). Unlike VS/UWS, several specific behavioral or perceptual functions are preserved in MCS (Stender et al., 2014). Accordingly, the difference in regional CMRglc between MCS and VS/UWS is found to be most pronounced in the frontoparietal and sensorimotor cortices whereas the subcortical structures are unaffected (Stender et al., 2015). Another signature of MCS, which is different from VS/UWS, is the local increase in cerebral glucose metabolism when the network is activated under external stimulation (Antal et al., 2011; Thibaut et al., 2015). The stimulation-induced widespread increase in the resting-state rCBF (~17.1%) suggests an increase in neural activities in a brain network that is functionally related to the stimulated area (Antal et al., 2011; Zheng et al., 2011). For patients with MCS, this response depends on residual brain metabolism in the DMN and thalamus and cortex integrity. Similarly, it is also true that sensory stimulation increases focal CMRglc under general anesthesia (Shulman et al., 1999). These results suggest that the response to an external stimulation requires a particular neuroenergetic level in both the thalamus and cortex, which is not found in individuals in a persistent VS.

**Figure 4:**
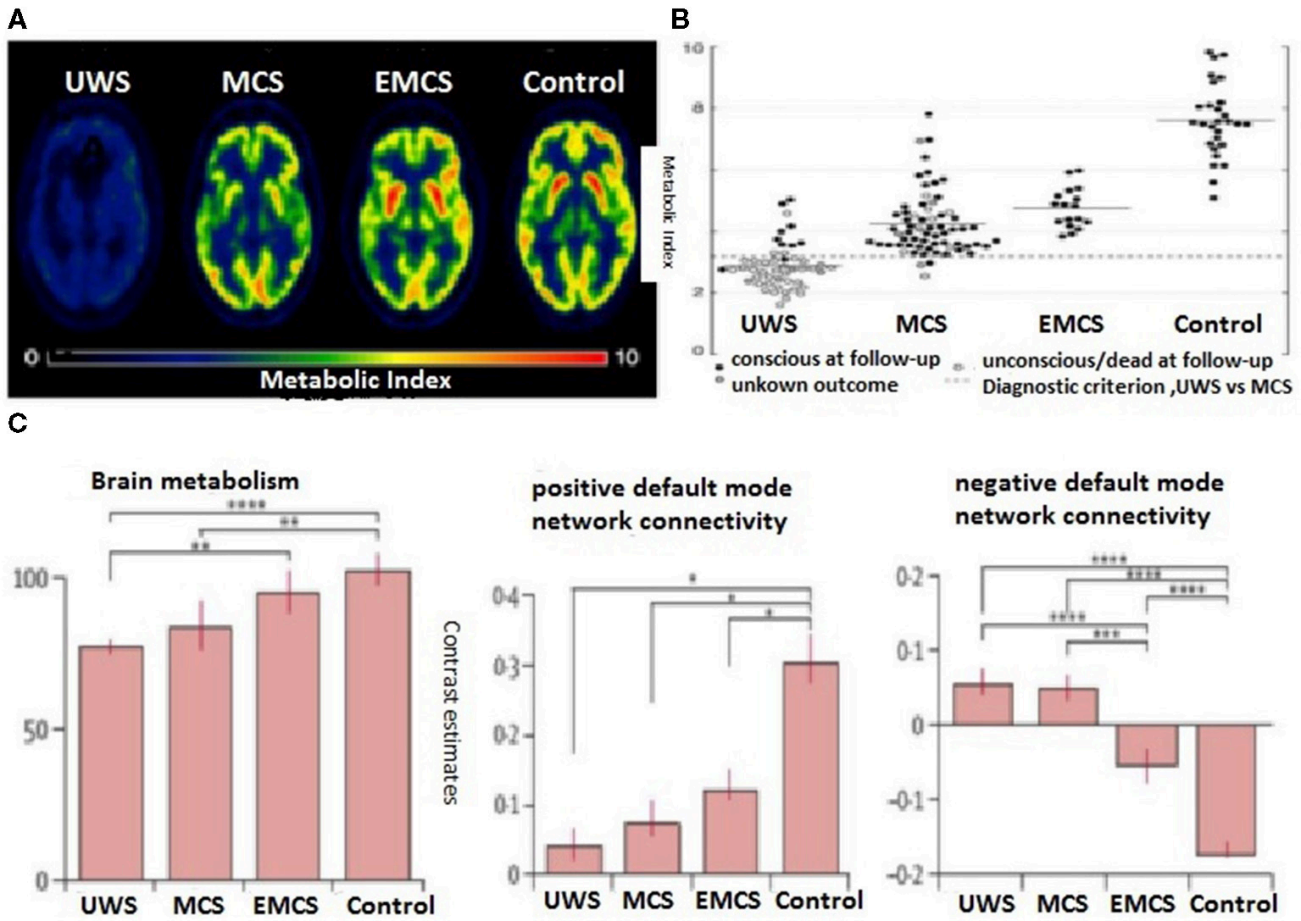
Individual global cerebral glucose metabolism using the FDG-PET measurement (metabolic index) in a patient following traumatic brain injury (TBI) with UWS, minimal conscious state (MCS), emergence from MCS, and health control. **(A)** Hypometabolism is the most prominent in patients with UWS (Stender et al., 2016). **(B)** Global brain glucose metabolism distribution of quantified cortical metabolic values for all individual subjects in the pooled cohort (Stender et al., 2016). **(C)** (Left) Between-group differences in positive and negative default-mode networks (DMNs) and brain metabolism. Positive DMN connectivity (within network correlations) was decreased, albeit preserved, in all patient groups, from vegetative state/unresponsive wakeful syndrome (VS/UWS), MCS, and emergence from MCS (EMCS) (Stender et al., 2016); no differences were identified between the groups of patients (middle). By contrast, patients differed in negative DMN connectivity (between network anticorrelations), and negative DMN connectivity only observed in patients who emerged from MCS and healthy controls (Stender et al., 2016) (right). Brain metabolism was more preserved in patients who had emerged from MCS than in patients with VS/UWS or MCS (Silva et al., 2010). *p < 0.05, **p = 0.01, ***p < 0.001, ****p < 0.0001.

The thalamus is considered to play a fundamental role in consciousness. In patients with DOCs, thalamic hypometabolism is most significant compared with healthy controls (Bruno et al., 2012). However, reduced thalamic glucose metabolism does not distinguish MCS from VS/UWS. Those DOC patients with remarkable reduced central thalamus metabolism also display a broad frontoparietal metabolic downregulation, and the recovery of frontoparietal metabolic activity is further associated with command following only (Fridman et al., 2014). These findings imply that the thalamus is not the single most important brain region determining DOC although it plays an essential role in arousal. The brainstem is another area of concern as the ascending arousal system is involved. Although brainstem metabolism is less affected than cortical metabolism, it declines to 60–70% of the normal average in both MCS and VS/UWS, suggesting that the brainstem is a necessary but not a sufficient contributor to consciousness. Taken together, these findings suggest that, although the patterns of neural activity in the subcortical system may be related to consciousness, metabolic rates in the primary sensorimotor areas, adjacent frontoparietal regions and precuneus, which are believed to integrate the input from frontoparietal networks and distinguish MCS (incomplete hypometabolism, 60% of normal) from VS/UWS (complete bilateral hypometabolism, 42% of normal) only (Figure 4). Thus, precuneal activity appears to tightly match general frontoparietal CMRglc. These data support global CMRglc as an indicator of consciousness while its regional variations reflect the integrity of specific perceptual or cognitive functions. This is consistent with the previous propositions (Fridman et al., 2014) that consciousness is associated with a whole-brain high energetic state rather than just metabolic activity in specific networks.

#### Cortical Networks and Connectivity in DOCs

As severe brain injury could disrupt the neural networks that sustain arousal and awareness, it is believed that FC decreases as a biomarker of consciousness level. This is exactly what seems to happen in deep sleep or under general anesthesia, alongside extensive frontoparietal hypometabolism. Similar decreases in FC, alongside the reduced levels of consciousness, are also observed in MCS and VS/UWS. PET and fMRI findings from resting-state- and stimulus-based studies suggest that a disruption of cortical connectivity affects multiple networks in DOC (Hannawi et al., 2015). However, recent evidence from patients with DOC also supports the view point that conscious awareness is associated with both cortical and subcortical connectivity in not only arousal-related networks but also those who are involved in awareness (Bodien et al., 2017). Furthermore, compared with healthy controls, patients with DOC exhibited fewer networks of neuronal origin, suggesting that the simultaneous disruption of connectivity in multiple networks rather than one single network leads to unconsciousness (Demertzi et al., 2014). In agreement with these findings, a significant similarity between the metabolic activity and FC from the FDG-PET/fMRI-based maps has also been demonstrated in patients with DOCs (Kirsch et al., 2017). PET studies in patients with VS/UWS have identified metabolic dysfunction in widespread frontoparietal networks, including midline DMN (“internal network”) and lateral frontoparietal associative cortices (“external network”; Annen et al., 2016; Soddu et al., 2016). This meta-analysis also demonstrated that reductions in neural or metabolic activity within midline anatomical structures in patients with DOCs were linked to the DMN (Hannawi et al., 2015). In contrast, metabolic disruption is shown in internal rather than external awareness networks (Thibaut et al., 2012), possibly contributing to their residual context-specific responsiveness to environmental demands in patients with MCS who display a better-preserved pattern of network connectivity (Crone et al., 2014).

Further analyses revealed that the DMN is metabolically preserved in patients who emerge from MCS rather than in patients with VS/UWS or patients remaining in MCS. Moreover, Di Perri et al. identified such a linkage between fMRI connectivity and cerebral glucose metabolism (Di Perri et al., 2016). The metabolic activity in the brain regions belonging to positive and negative DMN connectivity increases with progressive increments in consciousness levels, from VS/UWS, MCS, and the emergence from MCS to healthy controls (Figure 4). These metabolic changes, along with the conscious level-dependent decreases or increases in FC, corroborate the results of the previous studies supporting the energetic cost of FC.

Specifically, current evidence highlights the role of the posteromedial cortex (PMC) in regulating the consciousness levels in humans as a central hub between the networks (Crone et al., 2015; Silva et al., 2015; Zhang et al., 2017). The PMC, including the PCC and precuneus, is one of the most metabolically active brain regions. Selective hypometabolism in this structure has been reported in a wide range of altered consciousness states such as sleep (Horovitz et al., 2009) and anesthesia (Amico et al., 2014). Similarly, the impairment of glucose metabolism in the PCC (Kim et al., 2010) and precuneus (Silva et al., 2010) has been found in patients in a permanent VS. As long-distance connections are more energy-efficient, this hypometabolism would disrupt long-distance functional or MC between the ascending reticular activating system (ARAS) and PMC, and thus impairing access to conscious perception. In a recent study, Rosazza et al. showed that a higher metabolism in the precuneus has significantly stronger precuneus connectivity in MCS than VS/UWS (Rosazza et al., 2016). Conversely, elevated metabolism in PCC is associated with high positive and negative DMN connectivity in patients emerging from MCS, but not in unconscious patients, consistent with the notion that PCC is a crucial hub for consciousness.

Overall, although physiological, pharmacological, and pathological states of unconsciousness are poorly understood in terms of their underlying neural mechanisms, these results strongly suggest that functional integrity critically depends on energetic homeostasis within and between the brain networks important for conscious awareness. Understanding the relationship among the glucose metabolism, neural activity, and FC may help to improve the clinical outcomes in patients with consciousness disorders; for example, any interventions, including an external or internal stimulation, to increase the glucose metabolism in conscious networks may promote the neural activity, and thus promoting their FC, which in turn accelerates the patient recovery from anesthesia or DOC.

## Neuroenergetic Hypothesis of Consciousness

The brain is dynamically organized in space and time. The observations from physiological, pharmacological, and pathological states of unconsciousness suggest that brain activity and neuroglial glucose metabolism are directly linked as demonstrated in Shulman's studies (Shulman et al., 2003, 2014). We postulate that energy supports a spatiotemporal form of neural activity (FC) to generate consciousness. Once the energetic efficiency of the brain, maintaining homeostasis, declines, several high-energy-consuming neural correlates, such as complex reentrant thalamocortical dynamics, synchronization of gamma oscillation, glutamatergic synapses, or thalamic hubs, become deranged, leading to disruptions in global and local FC. Therefore, we hypothesize that brain metabolism enables energetic predispositions for the spatiotemporal organization of neural activities.

### Energetic-Metabolic Theory Supporting Consciousness

**Figure 5:**
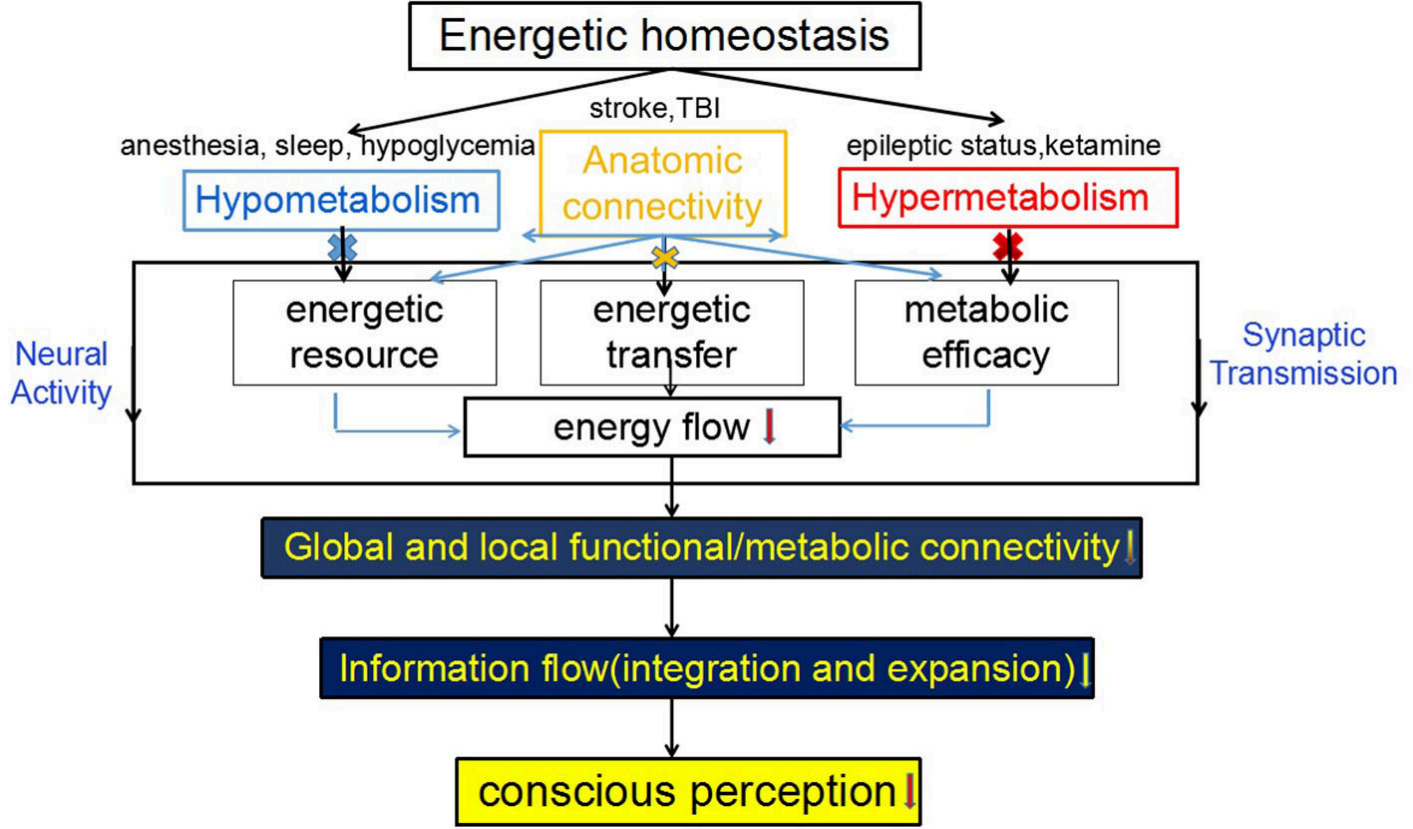
Schematic diagram of a coupling between energetic homeostasis and consciousness. The normal neuroglia metabolism maintains energetic homeostasis in the brain. The hypometabolism, hypermetabolism, or anatomic disconnection results from the reduced supply of energy substrate(s), metabolic efficacy or energetic transfer or communication, as a result, lower energy flow cannot drive neural activity and synaptic transmission, leading to the disruption of local and/or global functional/metabolic connectivity (MC), and the failure of information integration and globalization, and thereby the loss of consciousness.

A biological system is commonly homeostatic but not static. If it does not succeed in (re)establishing its balance, it may lead to the cessation of the functioning of the system. The dynamic system theory proposes that self-organization, energy flow, and state changes are central to a biological system (Smith, 2008) such as the brain. This self-organization in space and time is driven by the constant energy input, increasing the rate of energy transfer. The more ordered the complexity, the faster and broader the energy flows (Strelnikov, 2010). Using these general principles to study consciousness arising from the brain emphasizes the central role of neuroenergetics, facilitating the formation of the network wire connections into a self-organizing hierarchical system. As neural information processing entails energetic homeostasis to both operate and stabilize brain dynamics, a conscious state is likely to rely on neuroenergetic constraints as a means of optimizing the energy transfer rate (faster) and resource allocations (broader) to functional connection hubs in the case of anatomical integrity (Figure 5). For example, in an awake state, the brain dynamically optimizes the energy utilization coupled with the neural activity through extremely rapid global shifts in resource allocations (Schmidt et al., 2017). Theoretically, energetic homeostasis in an awake state is optimal for processing information because information processing (surrogated by gamma oscillation) decreases in both hypometabolic (anesthesia) and hypermetabolic (epilepsy) conditions. Both feedback and feedforward mechanisms work to ensure homeostasis. Recent studies showed that general anesthetics inhibit feedback (preferably) and feedforward neurotransmission in neural circuits (Sanders et al., 2018; Hudetz et al., 2020). Therefore, consciousness *per se* may be a result of homeostatic regulation. Evolutionally, sleep conserves energy by reducing the metabolic rate as an adaptation strategy with natural selection. Since the brain is the most energy-consuming organ, 10–20% decrements in CMRglc for energy conservation are sufficient to alter arousal levels. This energy conservation requires a structure capable of sensing and regulating energy status in the specific brain regions responsible for the awake/sleep cycle. The ventrolateral preoptic area (VLPO) may be one such candidate, as it is widely known not only as a key regulator of the sleep/wake cycle but also a sensor of energy status (Varin et al., 2015). This structure-function coupling seems to be well-designed. For example, regionally dynamic glucose levels in the VLPO determine the activation of one type of neuronal population while inhibiting others (Kong et al., 2010). In contrast, the neuronal energy load implies another mechanism whereby metabolites behave inversely on local excitatory/inhibitory activity to synchronize the input–output characteristics of specific neuron populations (Gordji-Nejad et al., 2018). These metabolism-dependent opposite effects may allow the network integration to modulate information flow with high-energy efficiency (Yu and Yu, 2017); as such, the output control of consciousness status is regulated by influencing EIB in a coordinated manner. Furthermore, energy homeostasis is likely to be an important constraint to generate complexity as metastable components, including supersaturated proteins and glutamate binding, are significantly enriched for synaptic processes and mitochondrial metabolism (Yu et al., 2018). Mitochondrial metabolism is crucial for generating fast cortical network rhythms through a precise synaptic transmission (Bas-Orth et al., 2020). Although mitochondria may adapt to enable energy homeostasis in response to an altered neuroglial energy state, substantial inhibition of mitochondrial function at synaptic sites affects a precise neurotransmission, contributing to information processing, and thereby consciousness (Rossi and Pekkurnaz, 2019). As an inherently unstable phenomenon, consciousness requires a continuous strict regulation by intrinsic energetic homeostasis; such metabolic mechanisms are also metastable in order to synchronize or coordinate between the brain networks to enable spatiotemporal globalization, expansion, or integration of spontaneous/evoked neural activity. The disruption to energy-dependent metastability in brain dynamics will be expected to adversely affect the spatiotemporal synchronization/coordination of internal/external information inputs, leading to the vulnerability of dynamic FC. Similar to systemic hypotension in which the blood flows slowly and redistributes itself to maintain perfusion to some organs (not the whole body) at the expense of other organs, the energy flow in an unconscious state may decline with the connectivity distance due to energy gradients from metabolic partitioning. Especially, energy flow during sleep or anesthesia may be confined to local areas and remain weak, rather than manifested in the form of global and strong connectivity, thereby potentially disrupting the global network complexity and coordination. Following energy flow normalization, information flow will be re-expanded and re-globalized across the whole brain, and global FC will return to normal. This assumption is derived from the fact that the interruption of energy flow may be the most economical way in terms of energy homeostasis for the transition between the conscious states, especially when FC binds again to the previous ones rather than creating an aberrant one, such that one can return to the original conscious state (including level and content) when awakening from anesthesia. For the pathological unconscious states, energy flow cannot be freely accessible between the networks undertaking high-order brain functions due to comprised anatomic integrity or regional energy. The brain must rebuild self-organizational processes of synaptic connectivity and energy-dependent imprinting of neural circuitry/networks if possible. Just as Lewis has indicated, the energy flow for self-organization is required to establish brain orders and may serve as a special type of “information” (Lewis et al., 1973). As such, unconscious patients regain consciousness when “energy-information coupling” is re-established and coordinated in a coherent spatial and temporal pattern (Kumaz, 2019). A recent theory based on thermodynamics indicates that neural networks in a normal brain naturally organize together according to energy costs into a sufficient number of connection “microstates” that lead to consciousness (Perez Velazquez et al., 2019). In summary, as the self-organization of neural activity in time and space is coupled and driven by energy flow once energetic homeostasis integrity is disrupted, the impairment of conscious-related information processing across the whole brain will lead to the loss of consciousness.

### Complementary Current Theories of Consciousness

**Figure 6:**
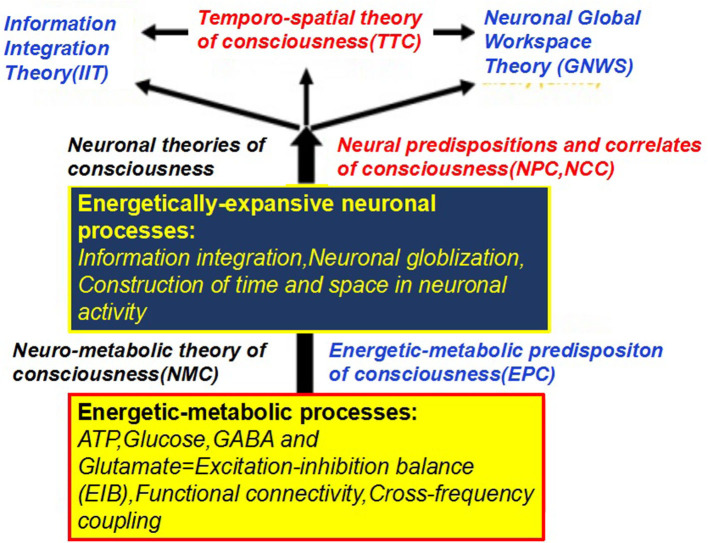
Neuro-metabolic theory of consciousness and its relationship to neuronal theories of consciousness. The figure shows in the lower part the energetic-metabolic basis of those neuronal processes (middle part) that are cost expensive and require lots of energic metabolisms to yield consciousness as postulated in various neuronal theories of consciousness (upper part).

Several popular theories of consciousness, including the IIT, GNWS, and TTC, have been presented with their own interpretations on consciousness. Separately, IIT posits that consciousness is based on information integration, which can be quantified mathematically according to the Phi metric (Casali et al., 2013). Experimentally, its operation requires an external perturbation such as transcranial magnetic stimulation (TMS). In the IIT context, the concept of information is defined as “*how a system of mechanisms in a state, through its cause-effect power, specifies a form in the space of possibilities”* rather than that traditionally understood in terms of specific contents. What proponents of IIT's describe as a space of possibilities may be closely related to the degree of available energetic-metabolic resource. Therefore, the concept of “*space of possibilities*” referring to different possible ways of information integration, as we assume, may be constrained by the energetic-metabolic resources. One would tentatively postulate both TMS-induced suppressions of ongoing activity and rest–stimulus interactions are correlated with a spatiotemporal expansion and integration of stimulus-evoked activity. As a spontaneous/evoked activity interaction responsible for switching on energy metabolism in neurons to increase their synaptic connectivity, we now hypothesize that such a spatiotemporal expansion and integration of internal/external stimuli, in general, requires expensive energetic supply. The more stimulus integrates and expands in spatiotemporal terms within the spontaneous activity, the more energy is required. Hence, what Tononi and the IIT describe as a “*space of possibilities”* may not only be a purely “*space of neuronal possibilities*,” but first and foremost an “*energetic-metabolic space of neuronal possibilities*.” The GNWS theory hypothesizes that consciousness emerges only when information processing gains access to the global workspace by attentional amplification leading to an “ignition” of neural circuits in the cerebral cortex. It propagates a widespread distribution of the information to other information processing events in the brain. Therefore, the GNWT theory postulates that consciousness allows for wider recruitment of neurons, and links information processing events across the modular networks, thus increasing the accessibility of information (conscious access). Compared with conscious processing, which involves widespread brain areas, unconscious processing can only be performed by limited brain areas. The architecture of the brain for globalizing and sharing stimulus-induced activity is supposed as a “*global workspace”* where the different modular networks responsible for various brain functions converge and overlap (“*spatial globalization*”). Similarly, the neural activities may extend to different temporal domains between the temporal components like event-related potentials (ERP) and gamma oscillation, which is defined as “*temporal globalization*.” Several high-energy-consuming neural correlates, such as complex re-enterant thalamocortical dynamics, and synchronization of gamma oscillation, glutamatergic synapses, or thalamic hubs, have been supposed to contribute to global access in the brain. As a more conscious perception involves more brain regions, we now postulate that conscious access of both “*spatial and temporal globalization*” of neural activity requires energy. Hence, the kind of neuronal, i.e., spatiotemporal globalization as envisioned in GNWS, may be based on sufficient energetic-metabolic resources. One could imagine the higher available energetic-metabolic resources the brain have, the greater ability spatiotemporal globalization of stimulus-induced activity has to be realized. In contrast, it may remain insufficient for subsequent spatiotemporal globalization, leading to the failure of conscious access. Finally, the TTC suggests that the different dimensions of consciousness, including content, level/state, and form (or organization), are temporal and spatial: they all presuppose the construction of time and space in the neuronal activity of the brain in a specific way (Northoff and Huang, 2017). Besides specific neuronal mechanisms that make it possible to construct time and space in neural activity, they first and foremost require energy: the more different frequencies or networks are nested within each other, the more energy is required. We, therefore, hypothesize that the brain metabolism provides the energetic-metabolic predispositions for temporospatial mechanisms. One may therefore want to speak of the energetic-metabolic predispositions of consciousness (EPC) that provide the necessary energetic-metabolic conditions of possible consciousness and its underlying neuronal-level temporospatial mechanisms. As such the EPC provides the energetic-metabolic underpinnings of the neural predispositions of consciousness (NPC) as well as NCC, including its enabling conditions (preNCC) and consequences (NCCcon). However, how energy is supplied and metabolized in different brain regions (spatially) and under different conscious states (temporally) is of high relevance in our understanding of consciousness. Overall, these theories emphasize that consciousness arises from the spatiotemporal expansion, integration, globalization, or organization of spontaneous/evoked neural activity. Although they are putative and have not been proven, some empirical evidence supports some of them more or less. In this study, we present a hypothesis as to how neuroenergetics provides a complementary perspective to these theories of consciousness (Figure 6), in which such a spatiotemporal expansion, integration, globalization, or organization, in general, requires a high energetic supply. The higher the available energetic resources the brain have, the greater the ability of the spatiotemporal configuration has to be realized, and thus generating conscious perception in the preservation of anatomic integrity. In contrast, if neuroenergetics remain insufficient for such a subsequent spatiotemporal configuration, the loss of consciousness would happen.

Nevertheless, according to IIT, if integrated energy in the whole brain is greater than that of the sum of all the neuronal cells, namely, a large enough value of integrated Phi, suggests this system is conscious. Obviously, neuroenergetic hypothesis emphasizes energy homeostasis rather than hypermetabolism in whole-brain or focal brain regions, such as during epilepsy and ketamine anesthesia, which does not guarantee the emergence of consciousness. Another example is that a hypermetabolism in the ARAS is found in a VS but FC between the ARAS and the precuneus is impaired (Silva et al., 2010), suggesting that hypermetabolism also does not guarantee functional integrity. In contrast, the GNWS theory posits that some “hubs” integrate information over space and time across an optimal number of connected neural networks. Although the brain consumes more energy to actively integrate and segregate the information from different senses in a conscious state than in an unconscious state; however, the conscious state is not about the total amount of energy in the brain but rather how the energy is organized. Therefore, our neuroenergetic hypothesis tends more to support the global nature of conscious awareness as advocated by the GNWS theory. Because neuroimaging studies showing a conscious perception seems to be associated with widespread cortical activity, especially in frontoparietal and medial temporal regions. As mentioned above, frontoparietal hypometabolism is implicated in the loss of consciousness induced by various causes, including deep sleep, coma, VS, Epilepsia, and general anesthesia. Another example is that the GNWS theory predicts that the damage to long-distance FC should impair access to conscious perception. The neuroenergetic hypothesis also indicates that the connectivity distance may decline with a decrease in the strength of energy flow. These findings are consistent with the GNWS theory, which is now favored by some neuroscientists.

## Conclusion

Thanks to classical investigations of Shulman focusing on the relationships between the brain metabolism and neural activity and neurotransmission, we have identified that normal energy metabolism may provide sufficient ATP to energize synaptic protein synthesis and maintain the information processing power of the brain. The latest study directly observed that the changes in neural activity were causally related to metabolic flux in intact circuits on the timescales associated with a behavior in drosophila brain (Mann et al., 2021), further convincing a large-scale tight coupling between the neural activity and energy metabolism across a brain network (Liu et al., 2013). Further, cerebral capillaries respond immediately to neuronal activation in an orchestrated network-level manner, supporting that local blood flow in the brain is tightly coupled to metabolic demands (Zhang et al., 2021). Therefore, our neuro-metabolic theory postulates that the special neuronal mechanisms underlying consciousness predispose specific energetic-metabolic resources. However, this does not mean that the mere presence of a high level of brain energy guarantees or entails the presence of consciousness. In addition to energetic-metabolic resources, consciousness requires specific neuronal mechanisms, such as information integration and/or neuronal globalization, based on the construction of brain of its own neuronal time and space. Therefore, our energetic-metabolic theory only describes a predisposition, not a neural correlate of consciousness; it may be complementary to the other theories of consciousness. Although mainstream neuroscience considers metabolism as a low-level domain, important but ultimately critical to information processing only when it goes wrong, it cannot exclude a more horizontal relationship between metabolism and neural signaling. As complex interactions between the energetic homeostasis and neural activity may integrate the correlates of consciousness, it is evident that the conscious states and neuroenergetics are not separable entities. In order to fully assess this hypothesis, integrating brain glucose metabolism and intrinsic functional activity with conscious states obtained in the same experimental setting may shed light on how a decoupling between metabolic cost and FC can contribute to the mechanisms underlying the loss of consciousness. We, furthermore, need to better understand the role of any other functional element known to affect energy resources and metabolic efficiency during transitions between the conscious states. The study of neuroenergetic dysfunction in the whole brain and regional brain areas is gaining interest in light of its ability to unravel the neural mechanisms of consciousness (Liu et al., 2013).

## Author Contributions

YC wrote the manuscript. JZ formed the idea and revised the manuscript. Both authors contributed to the article and approved the submitted version.

## Conflict of Interest

The authors declare that the research was conducted in the absence of any commercial or financial relationships that could be construed as a potential conflict of interest.
